# A Benzothiadiazole-Based Zn(II) Metal–Organic Framework with Visual Turn-On Sensing for Anthrax Biomarker and Theoretical Calculation

**DOI:** 10.3390/molecules29122755

**Published:** 2024-06-09

**Authors:** Jing Ru, Yi-Xuan Shi, Qing-Yun Yang, Teng Li, Hai-Ying Wang, Fan Cao, Qiang Guo, Yan-Lan Wang

**Affiliations:** 1 School of Chemistry and Chemical Engineering, Liaocheng University, Liaocheng 252059, China; 13954381561@163.com (Y.-X.S.); yangqy011011@163.com (Q.-Y.Y.); 13954002605@163.com (T.L.); wangyanlan@lcu.edu.cn (Y.-L.W.); 2 School of Environmental Science, Nanjing Xiaozhuang University, Nanjing 211171, China; 3 School of Materials Science and Engineering, Shandong Jianzhu University, Jinan 250101, China; caofan_success@126.com; 4 Institute of Biomedical Engineering, College of Life Sciences, Qingdao University, Ningxia Road 308, Qingdao 266071, China; guoqiang130@foxmail.com

**Keywords:** metal–organic framework, turn-on, biomarker, fluorescence sensor

## Abstract

2,6-pyridine dicarboxylic acid (DPA) is an exceptional biomarker of notorious anthrax spores. Therefore, the rapid, sensitive, and selective quantitative detection of DPA is extremely significant and urgent. This paper reports a Zn(II) metal–organic framework with the formula of {[Zn_6_(NDA)_6_(DPBT)_3_] 2H_2_O·3DMF}_n_ (MOF-1), which consists of 2,6-naphthalenedicarboxylic acid (2,6-NDA), 4,7-di(4-pyridyl)-2,1,3-benzothiadiazole (DPBT), and Zn(II) ions. Structural analysis indicated that MOF-1 is a three-dimensional (3D) network which crystallized in the monoclinic system with the *C*2/c space group, revealing high pH, solvent, and thermal stability. Luminescence sensing studies demonstrated that MOF-1 had the potential to be a highly selective, sensitive, and recyclable fluorescence sensor for the identification of DPA. Furthermore, fluorescent test paper was made to detect DPA promptly with color changes. The enhancement mechanism was established by the hydrogen-bonding interaction and photoinduced electron transfer transition between MOF-1 and DPA molecules.

## 1. Introduction

Biomarkers refer to biochemical indicators that can label structural or functional changes or potential changes in systems, organs, tissues, cells, and subcellular systems. The timely and reliable monitoring of biomarkers is crucial for preventing the outbreaks of related diseases [1,2]. As a unique component of anthrax spores, 2,6-Dipicolinic acid (DPA) is regarded as an appropriate biomarker for Bacillus spores [3]. Bacillus anthracis belongs to the aerobic Bacillus genus and can cause anthrax in animals such as sheep, cattle, horses, and humans. Bacillus anthracis spores have a tenacious vitality and can adapt to harsh environments such as vacuum, high temperature, and radiation [4]. According to reports, inhaling more than 10^4^ spores of Bacillus anthracis can lead to death without treatment with timely medication within 24–48 h [5]. Bacillus anthracis not only causes food poisoning and health problems, but is also a potential biological reagent [6,7]. Consequently, accurately identifying the DPA level is essential for preventing disease outbreaks and safeguarding homeland security. DPA quantification has been evaluated by Raman spectroscopy [8], polymerase chain reaction (PCR) [9], electrochemical methods [10], liquid chromatography [11], etc. However, the majority of the above methods are typically constrained by the inefficiencies of time-consumption pretreatment and high equipment costs.

Benefitting from their stable and various structures, tunable pore size, facile modification of pore surface, and intrinsic features, metal–organic frameworks (MOFs) have displayed great potential applications in gas adsorption, catalysis, drug delivery, organic molecule identification, and pollutant recognition [12,13,14,15,16,17,18]. In particular, a fluorescence MOF can effectively monitor target analytes in real time, which has attracted widespread attention in the field of biological systems [19,20,21,22]. Among them, the accurate and rapid monitoring of altered biochemical indicators in the content of biomarkers is of great significance for the diagnosis and even therapy of disease [23,24]. For example, a three-dimensional zinc(II)-MOF assembly by conjugated (E)-4,4′-(ethene-1,2-diyl)bis[(N-pyridin-3-yl)benzamide] and 1,3,5-benzenetricarboxylic acid was reported by Wang’s group. The Zn-MOF can be used as an effective fluorescence sensor for sensing the biomarker 3-nitrotyrosinea (3-NT) with a *K_SV_* value of 6.596 × 10^4^ M^−1^ [25]. Recently, Fan’s group proposed a Ni(II) metal–organic framework under a mixed-ligand method, and subsequently proved its excellent sensing properties in detecting 3-NT biomarker and HA biomarker in real samples [26]. As of now, “turn-off” responses (where fluorescence intensity decreases) are frequently used in documented examples to detect any analytes; on the contrary, “turn-on” responses (where fluorescence intensity increases) are uncommon [27]. Compared with “turn-off” responses, the signal change in fluorescence enhancement is easier to detect, and the fluorescence of the probe itself is weak, which can reduce the background signal and improve the sensitivity of the probe. At present, most fluorescence probes in applications belong to the type of enhanced fluorescence probes [28,29,30]. The above studies have not only verified the feasibility of planning MOF-based luminescence sensors as biomarkers, but also pointed out the direction for the next steps of research.

From the perspective of construction, multi-carboxylic organic ligands containing large π-conjugated aromatic structures and d^10^ metals are chosen to increase the diversity of MOF structures and the effectiveness of fluorescence recognition [31]. Firstly, π-conjugated organic linkers commonly provide the stability to form the metal–organic framework. Then, free π-electrons in the conjugated structure can freely move throughout the MOF network, reduce the HOMO-LUMO energy gap, and deliver attractive photophysical properties to the MOF. In addition, it is difficult for the d^10^ metal ions which contain a closed shell electronic configuration to realize the d–d electronic transition. The network formed from the d^10^ metal ions shows high luminescent intensity. This phenomenon could be attributed to the chelation of the metal ion by the ligand, which successfully enhances the rigidity of the ligand and diminishes the nonradiative decay. In short, to obtain MOFs with effective luminescent properties, Zn(II) and Cd(II) transition metal ions have been extensively adopted [32].

**Scheme 1:**
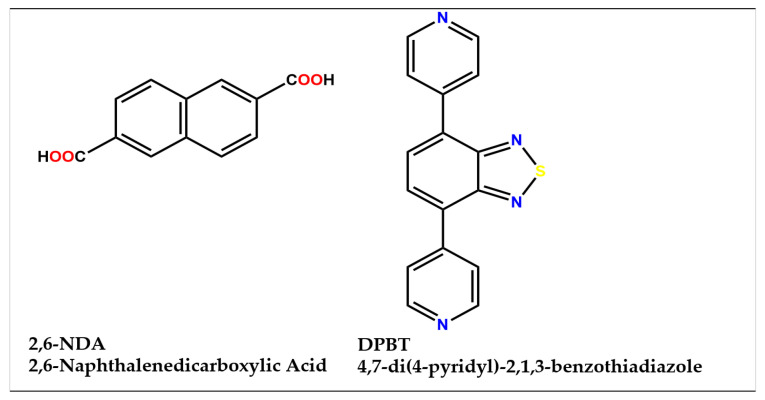
Molecular structures of 2,6-NDA and DPBT used for the construction of MOF-1.

In light of the aforementioned factors, we employed 2,6-naphthalenedicarboxylic acid (2,6-NDA), 4,7-di(4-pyridyl)-2,1,3-benzothiadiazole (DPBT) as the conjugated organic ligand, and d^10^ metal ions (Zn^2+^ ion) (Scheme 1). 2,1,3-benzothiadiazole (BTD) has unique photophysical chemical properties: (1) a large Stokes displacement; (2) a large molar extinction coefficient; (3) high quantum yield; and (4) when stored in solution or in pure solid form, high stability, where even after a long-time, irradiation will not fade. A series of benzothiadiazole-MOFs have been explored in recent years [33,34,35]. This paper successfully synthesized a luminescent MOF, {[Zn_6_(NDA)_6_(DPBT)_3_]·2H_2_O·3DMF}_n_ (MOF-1), using the solvothermal method. MOF-1 was fully characterized by means of X-ray single-crystal diffraction, thermogravimetric analysis (TGA), powder X-ray diffraction (PXRD), elemental analysis, and Fourier-transform infrared spectroscopy (FT-IR). According to the characterization analysis, MOF-1 showed good luminescence, water stability, and thermal stability. Luminescence tests illustrated that MOF-1 could be adopted as a turn-on response luminescent sensor for the detection of DPA in EtOH solution and fluorescence test papers. Furthermore, the mechanism of activating luminescence enhancement was discussed from a theoretical calculation perspective.

## 2. Results

### 2.1. X-ray Structure Determination

At room temperature, X-ray single-crystal data of MOF-1 were collected from a Siemens (Bruker) SMART CCD diffractometer with monochromated Mo K*α* radiation (*λ* = 0.71073 Å). The structure was solved using the direct method and refined by the full-matrix least-squares method on *F*^2^ with the SHELXTL software package [36,37]. PLATON [38] and SQUEEZE [39] were employed to calculate the diffraction contribution of the solvent molecules. Further crystallographic data and structural details are listed in Table 1 and Table S1, respectively. The CCDC number is 2324216 (MOF-1) (see Appendix A).

### 2.2. Crystal Structure of MOF-1

**Figure 1:**
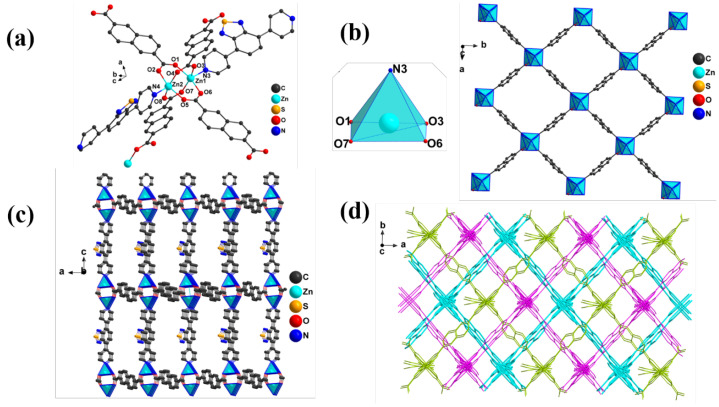
(**a**) The repeat unit MOF-1 (Zn_2_ center core); (**b**) coordination geometry of Zn(II) and 2D of MOF-1 through SBU and 2,6-NDA; (**c**) 3D of MOF-1 through DBPT along c axial; (**d**) interpenetrating 3D structure of MOF-1. (For clarity, the disordered and redundant atoms were omitted).

Structural analysis indicated that MOF-1 was a three-dimensional (3D) network, which crystallized in the monoclinic system with the *C*2/c space group. The binuclear Zn(II) ion was surrounded by two DPBT ligands and four deprotonated 2,6-NDA ligands, adopting bidentate bridge (μ_2_*-η*^1^*η*^1^*)* coordination mode (Figure 1a). The central Zn(III) ion was five-coordinated with a slightly irregular quadrangular pyramid coordination geometry occupied by eight oxygen atoms (O1, O3, O6, O7, N3) (Figure 1b). The bond distances of Zn–O/Zn-N were between 2.024 (7) and 2.066 (6) Å. The O(N)–Zn–O bond angles were located in the range of 87.0 (3) to 159.5 (3)°. The above data were similar to the values of the reported Zn-MOFs [40]. Furthermore, in the framework of MOF-1, the binuclear {Zn_2_(COO)_4_} as secondary building units (SBUs) with a non-bonding distance of Zn⋯Zn of 2.979 Å were observed (Figure 1c). Along the ab plane, SBUs were linked by four deprotonated 2,6-NDA ligands to form a two-dimensional (2D) structure, which was further connected by DPBT ligands to extend to a 3D network along c axial (Figure 1d). Within the framework, weak hydrogen bonding was observed between benzothiadiazole groups and protonated carboxylate groups of the ligands, such as C–H⋯O and C–H⋯N interactions.

### 2.3. Photoluminescence Properties

**Figure 2:**
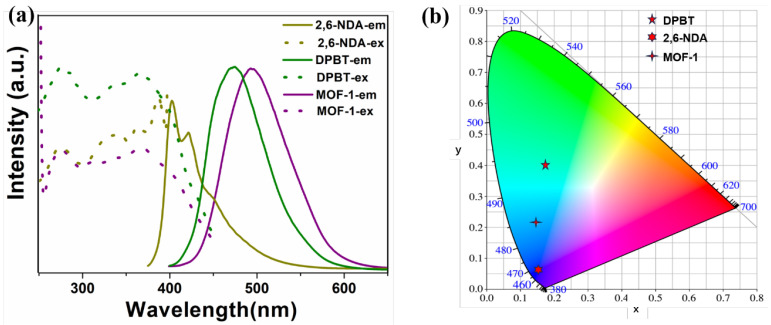
(**a**) The excitation and emission spectrum of solid-state 2,6-NDA, DPBT, and MOF-1 at room temperature; (**b**) CIE coordinates of the 2,6-NDA, DPBT, and MOF-1.

At room temperature, the luminescence properties of the free ligand 2,6-NDA, DPBT, and MOF-1 were thoroughly tested. As shown in Figure 2a, the carboxylate ligand 2,6-NDA exhibited prominent emission peaks with maximum values at 403 nm upon excitation at 362 nm, and the DPBT displayed emission peaks at 474 nm with λ_ex_ = 363 nm [41]. Compared with the emission peaks of free ligand 2,6-NDA and DPBT, the emissions peak of MOF-1 at 495 nm showed an obvious red-shift. This phenomenon should be attributed to the electron transition by coordination between O/N and Zn^2+^ in the framework [42]. Moreover, the CIE chromaticity diagrams of MOF-1, 2,6-NDA and DPBT are also drawn in Figure 2b. MOF-1, 2,6-NDA, and DPBT showed blue, purple, and green light with peaks of 495 nm, 403 nm, and 474 nm, respectively. In addition, the CIE coordinates of MOF-1, 2,6-NDA, and DPBT are (0.1470, 0.2142), (0.1548, 0.0641), and (0.1756, 0.3978), respectively. In addition, the emission peak of MOF-1 was 456 nm in ethanol solution, as shown in Figure S1.

### 2.4. FT-IR, PXRD analysis and Stability of MOF-1

The IR spectra of MOF-1 and the free ligands were examined in the range of 4000–400 cm^−1^ and shown in Figure S2. The characteristic peaks of 2,6-NDA at 1683 cm^−1^, 1425 cm^−1^ could be attributed to the asymmetric and symmetric vibrations of the C=O bonds. Compared with the IR absorption spectra of 2,6-NDA, the characteristic vibrational peaks in MOF-1 shifted to 1637 cm^−1^ and 1407 cm^−1^, suggesting that the -COOH group undergoes deprotonation to form the organometallic framework [43]. Furthermore, the observed peak at 1292 cm^−1^ vanished in MOF-1, verifying the coordination with Zn(II). This FT-IR spectra information was consistent with the behavior of the X-ray crystal structure.

**Figure 3:**
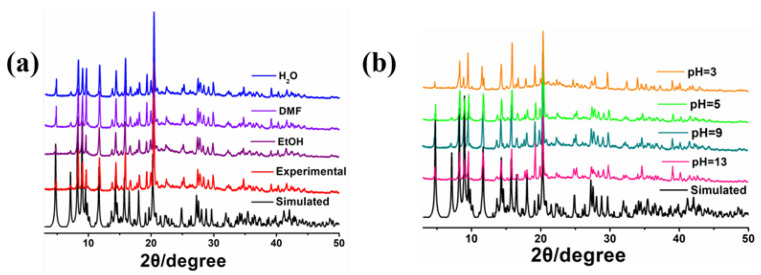
(**a**) PXRD patterns of MOF-1 after soaking in common solvent and (**b**) different pH aqueous solution for three days.

Powder X-ray diffraction (PXRD) analysis was used to characterize the purity and stability of the framework structure of MOF-1. The experimental data matched well with the simulated data, indicating that the obtained bulk sample was pure (Figure 3a). In order to evaluate the solvent and acid-base stabilities of MOF-1, the ground powder of the MOF-1 sample was soaked in common solvents and an acidic/alkaline aqueous solution (pH = 3, 5, 9, and 13) for 3 days, and the obtained PXRD remained in its original state (Figure 3a,b).

The thermal stability of MOF-1 was carried out by thermogravimetric analyzer (TGA) from 30 to 800 °C under a N_2_ atmosphere (Figure S3). The first weight loss of 9.25% (cal. 9.11%) was attributed to the reduction in uncoordinated solvent molecules (three DMF and two H_2_O) at 200 °C. The abrupt weight loss at about 400 °C could be induced by decomposition of organic ligands and the collapse of the framework, indicating the comparatively exceptional thermal stability of MOF-1.

### 2.5. Fluorescence Detection of DPA

**Figure 4:**
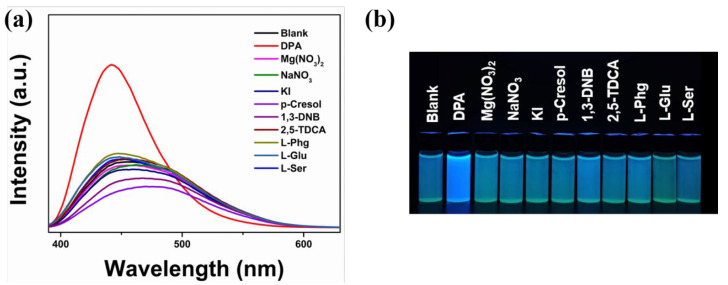
(**a**) The emission spectra of MOF-1 after dispersed in different analytes; (**b**) the corresponding color change in different analytes under UV light.

To precisely detect DPA and monitor the disease caused by Bacillus, luminescent experiments were carried out towards on MOF-1. Selectivity is an important characteristic of a luminescent sensor. Common ions or similar structure molecules Mg(NO_3_)_2_, NaNO_3_, Zn(NO_3_)_2_, KI, p-Cresol, 1,3-Dinitrobenzene (1,3-DNB), 2,5-thiophenedicarboxylic acid (2,5-TDCA), L-α-phenylglycine (L-Phg), glutamic acid (Glu), and L-Serine (L-Ser) were selected for the selectivity experiment. A 2 mg sample of MOF-1 was dispersed in ethanol with the 10^−3^ M analytes mentioned above. The luminescence intensities of MOF-1 were prominently dependent on the intrinsic features of the analytes (Figure 4a,b). Particularly, the DPA solution had a significant effect on the change in the fluorescence intensity of MOF-1, which greatly enhanced its fluorescence intensity. Compared with the blank emission of MOF-1 at 456 nm, a blue-shift of 13 nm from 456 nm to 443 nm was generated after being supplemented with DPA. On the contrary, no significant changes in the fluorescence spectra of MOF-1 were noticed in the presence of other analytes. According to this, MOF-1 could be a viable turn-on fluorescence sensor for accurately identifying DPA.

**Figure 5:**
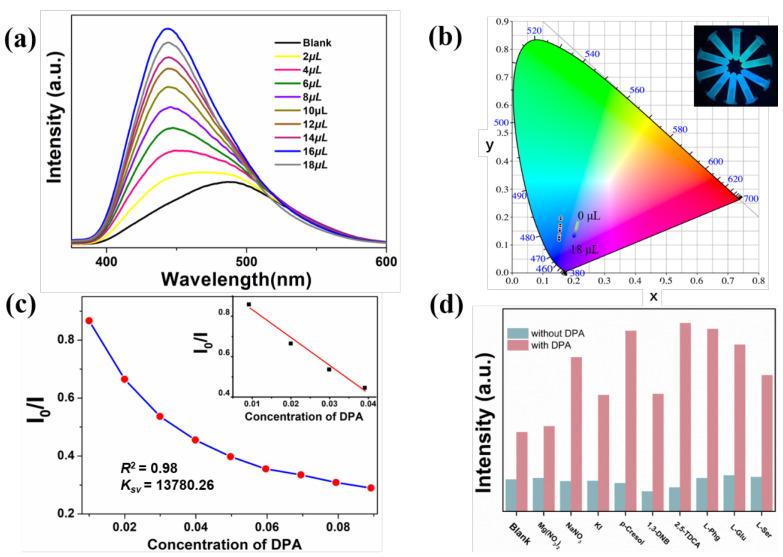
(**a**) Titration experiments of MOF-1 with different volume of DPA (0–18 μL, 10^−2^ M; (**b**) CIE chromaticity diagram showing the color coordinates of MOF-1 and MOF-1 containing DPA; (**c**) Stern–Volmer plots of MOF-1 for sensing of DPA and the linear relationship between *I*_0_*/I* and concentrations of DPA; (**d**) comparison of emission intensities of different analytes with and without DPA in MOF-1-EtOH solution.

Subsequently, the relationship between fluorescence intensity and DPA dosage was evaluated by quantitative experiments. As illustrated in Figure 5a, accompanied by adding DPA (1 × 10^−2^ M) to the suspension solution of MOF-1, the emission intensity showed a gradual increase and a blue shift. To further evaluate the emitting color change in MOF-1 toward DPA concentration, the CIE coordinates of the emission spectra of MOF-1 accompanied by an increase in DPA concentration were calculated (Figure 5b). The computed chromaticity gradually changes from green to blue, and there is a good consistency between DPA concentration and CIE coordinates. This could confirm the practicability of quantitatively detecting DPA by gauging the transformation in luminescent color. This change in solution fluorescence was also achieved through color changes observed with the naked eye under ultraviolet light (inset of Figure 5b). The relationship between the emission intensities of MOF-1 and the DPA concentration is illustrated in Figure 5c. At low DPA concentrations, the fluorescence enhancement curve can be quantitatively calculated with the Stern–Volmer equation (S-V): *I*_0_*/I* = 1+*K_sv_* [M] [44] (*I*_0_*/I* represents the ratio of emission intensity before and after DPA addition to MOF-1, [M] represents the concentration of DPA, and *K_sv_* represents the fluorescence enhancement constant). As we expected, the detection of DPA had a satisfactory linear relationship (R^2^ > 0.98), and the enhancement constant (*K_sv_*) of MOF-1 was 1.38 × 10^4^ M^−1^. Meanwhile, the detection limit was determined by the equation (LOD): LOD = 3σ/K (σ: calculate the standard deviation of 10 standard blank samples), and the corresponding LOD was 0.025 μM.

It is worth noting that a few examples of MOF-based luminescent chemical sensors for sensing DPA have been reported. To compare with the reported MOFs, the classic sensors are listed in Table S2 [45,46,47]. Considering the significant complexity of the samples detected in practical applications, the anti-interference sensing ability of MOF-1 was determined through competitive experiments. The experiments have shown that in the presence of other interfering substances, the emission spectra and fluorescent test strip detection still have good recognition effects on DPA (Figure 5d and Figure S4).

### 2.6. Recyclability and Visualizable Sensing

**Figure 6:**
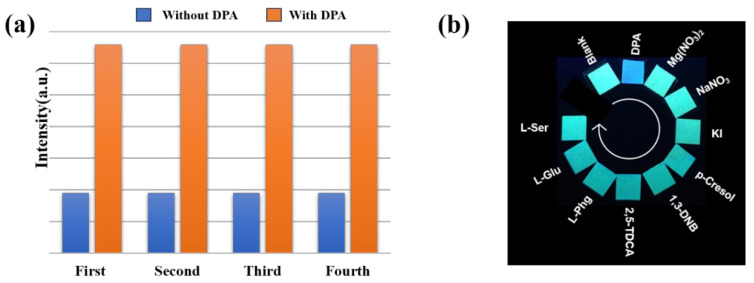
(**a**) The reversibility of MOF-1 for DPA sensing; (**b**) test paper of different analytes under UV light.

The cyclic performance of sensing is an important prerequisite for a good fluorescence sensor in practical applications. As shown in Figure 6a, after numerous times washing with DMF and EtOH, the sensing experiment proved that the fluorescence intensity of the processed MOF-1 can be restored to its original intensity. The aforementioned tests provided evidence that MOF-1 may function as a promising material with exceptional economy for DPA sensing.

Fluorescent test papers can not only respond more quickly, but also allow for easier visualization. Hence, fluorescent test strips were prepared by immersing the filter paper in MOF-1 suspension and ultrasonic treatment for 30 min. Compared with blank fluorescent test strips, the presence of DPA can be revealed through color changes when irradiated with a 365 nm UV lamp. As depicted in Figure 6b, only the test paper titrated with DPA showed a significant color change, which clearly supported the high selectivity of MOF-1 for DPA. This change provided the possibility for rapid and simple testing with the naked-eye detection of DPA in ethanol solution.

## 3. Discussion

### Mechanism of Luminescence Enhancing

To gain a deeper understanding of the DPA turn-on response mechanism, further experiments including PXRD, UV-vis, FT-IR, and DFT calculation were arranged. Firstly, the PXRD patterns and IR spectra remain unchanged after immersing in DPA for 24 h, excluding the reasons of crystal collapse and disintegration during the sensing process (Figures S5 and S6). Then, Figure S7 demonstrates a slight overlap between the emission bands/excitation bands of MOF-1 and the UV–vis absorption spectra of DPA, indicating that the reason for the fluorescence-enhanced response of MOF-1 to DPA is not mainly due to competitive absorption mechanisms or Förster resonance energy transfer mechanisms [48].

**Figure 7:**
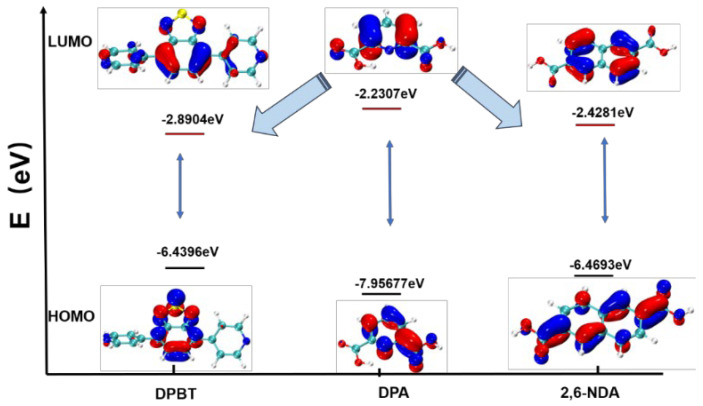
The HOMO, LUMO, and HOMO–LUMO energy band gaps for DPBT, DPA, and 2,6-NDA.

According to earlier reports, one reason for the chemo-sensor for fluorescence quenching is that the energy of the lowest unoccupied molecular orbital (LUMO) of the analyte falls within the valence band (VB) and conduction band (CB) of the chemical sensor. When the fluorescence intensity is enhanced due to the transfer of electrons from the chemical sensor CB to the LUMO of the analyte, a clear contrast phenomenon can be observed. Therefore, the Gaussian 16 program [49] was used to calculate the molecular orbitals of 2,6-NDA, DPBT, and DPA. Single-point calculation and optimization were carried on the level of B3LYP/def2-SVP and B3LYP/def2-TZVP [50,51], considering the DFT-D3 (BJ) dispersion correction [52] and the SMD solvation model. The molecular orbitals were analyzed and mapped by VMD 1.9.4 [53] combined with Multiwfn 3.8(dev) software [54]. As shown in Figure 7, the LUMO (−2.43 eV) of 2,6-NDA and (−2.89 eV) of DPBT are lower than the LUMO (−2.36 eV) of DPA. As a result, there will be an electron transfer from the LUMO of DPA to the LUMO of the linker, resulting in the observed “turn-on” of luminescence. The lifetime decay experiment can further promote researchers’ understanding of the recognition mechanism. The fluorescence lifetime of MOF-1 is 0.98 ns, and after adding DPA, the lifetime value of MOF-1 increases to 6.09 ns. In addition, the fluorescence amplification curve of MOF-1 (EtOH), MOF-1 (EtOH) @18 μM DPA can be observed (Figure S8), and this series of data show the same intensification trend, indicating that this is a dynamic turn-on mechanism.

**Figure 8:**
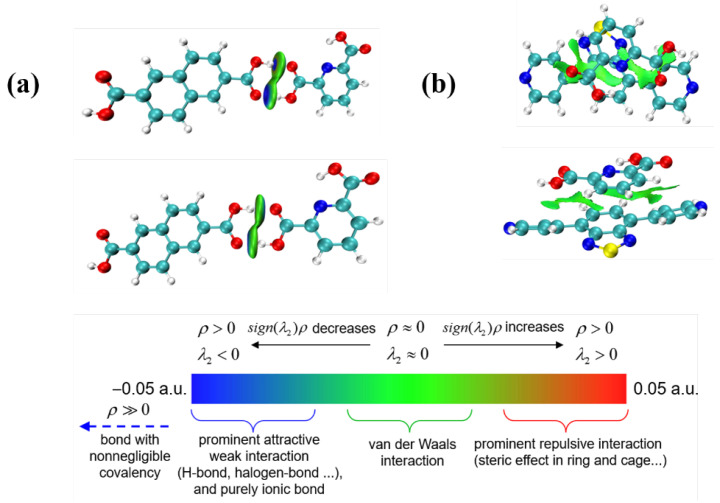
(**a**) Optimized geometry of the 2,6-NDA ligand with DPA and a gradient isosurface; (**b**) optimized geometry of the DPBT ligand with DPA and a gradient isosurface.

The red shift observed in the fluorescence emission indicates substantial exciplex formation between MOF-1 and DPA. Compared to fresh MOF-1, there is neither the appearance of new bands nor the disappearance of existing bands in the IR spectrum, which were measured by MOF-1 after soaking in DPA solution. This indicates that there is no coordination between DPA and MOF-1. Considering the porosity of MOF-1, these phenomena are different from the common guest-induced sensing mechanism, which may be caused by some interactions between MOF-1 and DPA absorbed on the surface of MOF-1. The independent gradient model based on Hirshfeld partition (IGMH) [55] using the VMD program was used to evaluate the weak intermolecular interactions such as van der Waals distance, hydrogen bonding, and steric effects between DPA molecules and 2,6-NDA/DPBT ligands. By distinguishing different weak interactions through color, it can be observed that strong hydrogen bonds are represented by the blue part between the optimized structures of 2,6-NDA ligands with DPA (Figure 8a), while in the optimized structures of DPBT ligands and DPA, the van der Waals distance is represented by the green part (Figure 8b). The DFT result was further supported by the link between absorbance and DPA titration. The UV–vis absorption value of MOF-1 gradually increases with the addition of different amounts of DPA (Figure S9), which further illustrates the interaction between the DPA and the MOF-1 structure. That is to say, the hydrogen bonding between DPA and the 2,6-NDA ligands can increase affinity and easily accept electrons, which is beneficial for the charge flow in the ligand-to-ligand charge transfer process, leading to a turn-on response at 443 nm.

Overall, the noticeable enhancement in the emission intensity and the red shift of the emission wavelength suggested the possibility of electron transfer from the LUMO of DPA to MOF-1, together with the formation of hydrogen bonding interactions between DPA and MOF-1.

## 4. Materials and Methods

### 4.1. Reagents and Methods

2,6-NDA, DPBT, N, N-Dimethylformamide (DMF) and other organic reagents (analytical grade) were purchased from Bide Pharmatech Ltd. (Shanghai, China). Zn(NO_3_)_2_ was purchased from Aladdin Bio Chem Technology Co. Ltd. (Shanghai, China). The raw materials were commercially purchased and did not require purification. NICOLET 5700F-IR spectrometer (Shanghai, China) was used to measure the infrared spectrum in the range of 4000–400 cm^−1^. Elemental analyses were examined on a PE 2400 II analyzer. Under a N_2_ atmosphere, thermal gravimetric (TG) data were collected and recorded on a Netzsch STA-449F5 (Selbu, Germany) thermo analyzer with heating rate of 10 °C/min. Powder X-ray diffraction (PXRD) was carried out using a Bruker D8 Advance diffractometer with Cu-Kα radiation at room temperature. Fluorescence decay lifetime was recorded on an Edinburgh FLS 1000 fluorescence spectrometer (Livingston, UK). All the fluorescence spectrum tests were implemented with a Hitachi F-7000 spectrometer (Tokyo, Japan).

### 4.2. Synthesis of MOF-1

DPBT (2.9 mg, 0.010 mmol), 2,6-NDA (2.16 mg, 0.010 mmol), Zn(NO_3_)_2_·6H_2_O (11.90 mg, 0.04 mmol), and HNO_3_ (2 drops, 2 M) were successively added into a Teflon vessel with a mixture of *V_DMF_/V_H2O_/V_CH3CN_* (3:4:1). After ultrasound for 30 min, the above mixture was sealed and heated to 100 ℃ for one day. After cooling, yellow rodlike crystals were obtained. Yield: 62% based on 2,6-NDA ligand. IR Spectra (KBr, m/cm^−1^): 3430 s, 2923 s, 1670 s, 1637 s, 1552 w, 1407 s, 1361 s, 1223 w, 1089 s, 1032 w, 822 m, 792 s, 482 s.

## 5. Conclusions

In summary, one Zn(II)-MOF (MOF-1) based on 2,6-naphthalenedicarboxylic acid (2,6-NDA) and 4,7-di(4-pyridyl)-2,1,3-benzothiadiazole was effectively synthesized by a mixed-ligand approach. MOF-1 exhibited high purity, pH stability, and thermal stability. Luminescent experiments demonstrated that MOF-1 could be employed as a selective, sensitive, and convenient turn-on response sensor for DPA detection. In addition, based on the results of PXRD, UV–vis, FT-IR, XPS, and theoretical calculations, the enhancement mechanism between MOF-1 and DPA could be summarized by photoinduced electron transfer and hydrogen bonding interactions. The visualization of the fluorescent test paper makes it possible for the practical application of MOF-1. More biomarker sensors will be fabricated by our group in the future.

## Figures and Tables

**Table 1:** Crystallographic data for the MOF-1.

	MOF-1
Empirical formula	C_120_H_64_N_12_O_24_S_3_Zn_6_
Formula weight	2546.23
Crystal system	monoclinic
Space group	C2/c
a, Å	17.6526 (17)
b, Å	19.6903 (19)
c, Å	37.142 (3)
α, deg	90
β, deg	90.106 (2)
γ, deg	90
V, Å^3^	12,910 (2)
Z	4
*ρ*_calcd_, g/cm^−3^	1.310
T/K	298.15
*μ*, mm^−1^	1.214
2*θ*, deg	3.794 to 50.038
F (000)	5152.0
Index ranges	−20 ≤ h ≤ 20, −23 ≤ k ≤ 23, −26 ≤ l ≤ 44
Data/restraints/parameters	11,387/2403/779
GOF (F^2^)	1.067
*R_1_^a^*,*wR_2_^b^* (I > 2σ(I))	0.1070, 0.2790
*R_1_^a^*, *wR_2_^b^* (all data)	0.1591, 0.3075

*R*_1_*^a^* = Σ||*F*_o_| − |*F*_c_||/Σ*F*_o_|. *wR*_2_*^b^* = [Σ*w*(*F*_o_^2^ − *F*_c_^2^)^2^/Σ*w*(*F*_o_^2^)^2^]^1/2^.

## Data Availability

Details are available from the authors.

## Supplementary Materials

The following supporting information can be downloaded at: https://www.mdpi.com/article/10.3390/molecules29122755/s1, Figure S1: the excitation and emission of MOF-1 in EtOH; Figure S2: IR of MOF-1 and organic ligand 2,6-NDA; Figure S3: the TGA curve of MOF-1 under N_2_ atmosphere from 30 to 800 °C; Figure S4. interference experiments of different analytes with and without DPA-test strips (a) and the emission spectrum curves (b). Figure S5: the PXRD pattern of MOF-1-and the sample after sensing DPA; Figure S6: IR of MOF-1 after sensing of DPA; Figure S7: absorption spectra of DPA and emission bands/excitation of MOF-1; Figure S8: fluorescence lifetime of MOF-1 before and after DPA; Figure S9: absorption spectra of MOF-1 dispersed in EtOH solution after adding different concentrations of DPA; Table S1: selected bond lengths (Å) and angles (°) for MOF-1; Table S2: comparison of the literature reports for MOFs as sensors of DPA.

## Appendix A

CCDC 2324216 contains the supplementary crystallographic data for this paper. The data can be obtained free of charge via http://www.ccdc.cam.ac.uk/ (accessed on 30 April 2024) or by emailing data_request@ccdc.cam.ac.uk.
