# Supplementary material for: A Benzothiadiazole-Based Zn(II) Metal–Organic Framework with Visual Turn-On Sensing for Anthrax Biomarker and Theoretical Calculation
Source: Molecules. 2024 Jun 9;29(12):2755. doi: 10.3390/molecules29122755 (PMC11206062; doi:10.3390/molecules29122755)

checkCIF/PLATON report

Structure factors have been supplied for datablock(s) 230303f_sq

THIS REPORT IS FOR GUIDANCE ONLY. IF USED AS PART OF A REVIEW PROCEDURE FOR PUBLICATION, IT SHOULD NOT REPLACE THE EXPERTISE OF AN EXPERIENCED CRYSTALLOGRAPHIC REFEREE.

No syntax errors found.   CIF dictionary   Interpreting this report

Datablock: 230303f_sq

Bond precision:	C-C = 0.0153 A		Wavelength=0.71073
Cell:	a=17.6526(17)	b=19.6903(19)	c=37.142(3)
	alpha=90	beta=90.106(2)	gamma=90
Temperature:	298 K		

	Calculated	Reported
Volume	12910(2)	12910(2)
Space group	C 2/c	C 1 2/c 1
Hall group	-C 2yc	-C 2yc
Moiety formula	2(C40 H21 N4 O8 S Zn2), C40 H22 N4 O8 S Zn2 [+ solvent]	C120 H64 N12 O24 S3 Zn6
Sum formula	C120 H64 N12 O24 S3 Zn6 [+ solvent]	C120 H64 N12 O24 S3 Zn6
Mr	2546.35	2546.23
Dx,g cm-3	1.310	1.310
Z	4	4
Mu (mm-1)	1.214	1.214
F000	5152.0	5152.0
F000'	5162.27	
h,k,lmax	20,23,44	20,23,44
Nref	11406	11387
Tmin,Tmax	0.852,0.941	0.852,0.941
Tmin'	0.654	

Correction method= # Reported T Limits: Tmin=0.852 Tmax=0.941
AbsCorr = NONE

Data completeness= 0.998   Theta(max)= 25.019

```
R(reflections)= 0.1070( 6426)                wR2(reflections)=
                                             0.3075( 11387)
S = 1.067              Npar= 779
```

The following ALERTS were generated. Each ALERT has the format
test-name_ALERT_alert-type_alert-level.
Click on the hyperlinks for more details of the test.

Alert level B

```
PLAT341_ALERT_3_B Low Bond Precision on  C-C Bonds ..............    0.01535 Ang.
```

Alert level C

```
PLAT082_ALERT_2_C High R1 Value ................................       0.11 Report
PLAT084_ALERT_3_C High wR2 Value (i.e. > 0.25) ..................       0.31 Report
PLAT242_ALERT_2_C Low    'MainMol' Ueq as Compared to Neighbors of       Zn1 Check
PLAT242_ALERT_2_C Low    'MainMol' Ueq as Compared to Neighbors of       Zn2 Check
PLAT242_ALERT_2_C Low    'MainMol' Ueq as Compared to Neighbors of       Zn3 Check
PLAT410_ALERT_2_C Short Intra H...H Contact  H3        ..H8       .       1.96 Ang.
                                                     x,y,z  =      1_555 Check
PLAT906_ALERT_3_C Large K Value in the Analysis of Variance ......      5.421 Check
PLAT906_ALERT_3_C Large K Value in the Analysis of Variance ......      2.714 Check
PLAT906_ALERT_3_C Large K Value in the Analysis of Variance ......      2.028 Check
PLAT911_ALERT_3_C Missing FCF Refl Between Thmin & STh/L=    0.595         15 Report
PLAT934_ALERT_3_C Number of (Iobs-Icalc)/Sigma(W) > 10 Outliers ..          1 Check
PLAT971_ALERT_2_C Check Calcd Resid. Dens.  1.28Ang From C11             1.63 eA-3
PLAT972_ALERT_2_C Check Calcd Resid. Dens.  0.10Ang From S2             -1.88 eA-3
PLAT972_ALERT_2_C Check Calcd Resid. Dens.  0.18Ang From S2'            -1.83 eA-3
PLAT972_ALERT_2_C Check Calcd Resid. Dens.  0.46Ang From C11            -1.52 eA-3
PLAT975_ALERT_2_C Check Calcd Resid. Dens.  0.94Ang From O4       .      0.79 eA-3
PLAT975_ALERT_2_C Check Calcd Resid. Dens.  0.97Ang From O3       .      0.69 eA-3
PLAT977_ALERT_2_C Check Negative Difference Density on H44        .     -0.32 eA-3
PLAT977_ALERT_2_C Check Negative Difference Density on H47'       .     -0.34 eA-3
```

Alert level G

```
PLAT002_ALERT_2_G Number of Distance or Angle Restraints on AtSite          5 Note
PLAT003_ALERT_2_G Number of Uiso or Uij Restrained non-H Atoms ...         89 Report
PLAT004_ALERT_5_G Polymeric Structure Found with Maximum Dimension          3 Info
PLAT007_ALERT_5_G Number of Unrefined Donor-H Atoms .............          4 Report
PLAT042_ALERT_1_G Calc. and Reported MoietyFormula Strings  Differ     Please Check
PLAT066_ALERT_1_G Predicted and Reported Tmin&Tmax Range Identical          ? Check
PLAT072_ALERT_2_G SHELXL First  Parameter in WGHT  Unusually Large       0.16 Report
PLAT083_ALERT_2_G SHELXL Second Parameter in WGHT  Unusually Large      64.37 Why ?
PLAT171_ALERT_4_G The CIF-Embedded .res File Contains EADP Records          4 Report
PLAT172_ALERT_4_G The CIF-Embedded .res File Contains DFIX Records          6 Report
PLAT174_ALERT_4_G The CIF-Embedded .res File Contains FLAT Records          1 Report
PLAT178_ALERT_4_G The CIF-Embedded .res File Contains SIMU Records          1 Report
PLAT188_ALERT_3_G A Non-default SIMU Restraint Value has been used     0.0100 Report
PLAT301_ALERT_3_G Main Residue  Disorder .............(Resd  2  )        13% Note
PLAT343_ALERT_2_G Unusual sp?     Angle Range in Main Residue for        C10 Check
PLAT367_ALERT_2_G Long?  C(sp?)-C(sp?) Bond  C10       - C11      .       1.51 Ang.
PLAT432_ALERT_2_G Short Inter X...Y Contact  S2        ..C10      .       2.21 Ang.
                                                1-x,1-y,-z  =      5_665 Check
```

```
PLAT432_ALERT_2_G Short Inter X...Y Contact  S2       ..C11      .       2.37 Ang.
                                                 1-x,1-y,-z  =      5_665 Check
PLAT432_ALERT_2_G Short Inter X...Y Contact  S2'      ..C10      .       3.23 Ang.
                                                      x,y,z  =      1_555 Check
PLAT432_ALERT_2_G Short Inter X...Y Contact  S2'      ..C11      .       3.30 Ang.
                                                      x,y,z  =      1_555 Check
PLAT432_ALERT_2_G Short Inter X...Y Contact  C11      ..N6       .       3.03 Ang.
                                                 1-x,1-y,-z  =      5_665 Check
PLAT606_ALERT_4_G Solvent Accessible VOID(S) in Structure ........          ! Info
PLAT779_ALERT_4_G Suspect or Irrelevant (Bond) Angle(s) in CIF ...      18.10 Deg.
              N6   -C47  -H47     1_555   1_555   1_555 ......     #  252 Check
PLAT779_ALERT_4_G Suspect or Irrelevant (Bond) Angle(s) in CIF ...       6.50 Deg.
              N7   -C48  -H48     1_555   1_555   1_555 ......     #  259 Check
PLAT779_ALERT_4_G Suspect or Irrelevant (Bond) Angle(s) in CIF ...      30.20 Deg.
              N6'  -C47' -H47'    1_555   1_555   1_555 ......     #  264 Check
PLAT779_ALERT_4_G Suspect or Irrelevant (Bond) Angle(s) in CIF ...      30.50 Deg.
              N7'  -C48' -H48'    1_555   1_555   1_555 ......     #  269 Check
PLAT779_ALERT_4_G Suspect or Irrelevant (Bond) Angle(s) in CIF ...      30.30 Deg.
              C47  -N6   -H47     1_555   1_555   1_555 ......     #  270 Check
PLAT779_ALERT_4_G Suspect or Irrelevant (Bond) Angle(s) in CIF ...      16.20 Deg.
              C48  -N7   -H48     1_555   1_555   1_555 ......     #  273 Check
PLAT779_ALERT_4_G Suspect or Irrelevant (Bond) Angle(s) in CIF ...      43.50 Deg.
              C47' -N6'  -H47'    1_555   1_555   1_555 ......     #  276 Check
PLAT779_ALERT_4_G Suspect or Irrelevant (Bond) Angle(s) in CIF ...      18.00 Deg.
              N6   -S2   -H47     1_555   1_555   1_555 ......     #  286 Check
PLAT779_ALERT_4_G Suspect or Irrelevant (Bond) Angle(s) in CIF ...      12.10 Deg.
              N7   -S2   -H48     1_555   1_555   1_555 ......     #  291 Check
PLAT779_ALERT_4_G Suspect or Irrelevant (Bond) Angle(s) in CIF ...      22.00 Deg.
              N7'  -S2'  -H48'    1_555   1_555   1_555 ......     #  294 Check
PLAT794_ALERT_5_G Tentative Bond Valency for Zn1       (II)      .       2.00 Info
PLAT794_ALERT_5_G Tentative Bond Valency for Zn2       (II)      .       1.97 Info
PLAT794_ALERT_5_G Tentative Bond Valency for Zn3       (II)      .       1.98 Info
PLAT860_ALERT_3_G Number of Least-Squares Restraints .............       2403 Note
PLAT868_ALERT_4_G ALERTS Due to the Use of _smtbx_masks Suppressed          ! Info
PLAT910_ALERT_3_G Missing # of FCF Reflection(s) Below Theta(Min).          4 Note
PLAT941_ALERT_3_G Average HKL Measurement Multiplicity ...........        2.7 Low
PLAT967_ALERT_5_G Note: Two-Theta Cutoff Value in Embedded .res ..       50.0 Degree
PLAT978_ALERT_2_G Number C-C Bonds with Positive Residual Density.          0 Info
```

```
   0 ALERT level A = Most likely a serious problem - resolve or explain
   1 ALERT level B = A potentially serious problem, consider carefully
  19 ALERT level C = Check. Ensure it is not caused by an omission or oversight
  41 ALERT level G = General information/check it is not something unexpected

   2 ALERT type 1 CIF construction/syntax error, inconsistent or missing data
  25 ALERT type 2 Indicator that the structure model may be wrong or deficient
  12 ALERT type 3 Indicator that the structure quality may be low
  16 ALERT type 4 Improvement, methodology, query or suggestion
   6 ALERT type 5 Informative message, check
```

It is advisable to attempt to resolve as many as possible of the alerts in all categories. Often the minor alerts point to easily fixed oversights, errors and omissions in your CIF or refinement strategy, so attention to these fine details can be worthwhile. In order to resolve some of the more serious problems it may be necessary to carry out additional measurements or structure refinements. However, the purpose of your study may justify the reported deviations and the more serious of these should normally be commented upon in the discussion or experimental section of a paper or in the "special_details" fields of the CIF. checkCIF was carefully designed to identify outliers and unusual parameters, but every test has its limitations and alerts that are not important in a particular case may appear. Conversely, the absence of alerts does not guarantee there are no aspects of the results needing attention. It is up to the individual to critically assess their own results and, if necessary, seek expert advice.

Publication of your CIF in IUCr journals

A basic structural check has been run on your CIF. These basic checks will be run on all CIFs submitted for publication in IUCr journals (*Acta Crystallographica*, *Journal of Applied Crystallography*, *Journal of Synchrotron Radiation*); however, if you intend to submit to *Acta Crystallographica Section C* or *E* or *IUCrData*, you should make sure that full publication checks are run on the final version of your CIF prior to submission.

Publication of your CIF in other journals

Please refer to the *Notes for Authors* of the relevant journal for any special instructions relating to CIF submission.

PLATON version of 06/07/2023; check.def file version of 30/06/2023

Datablock 230303f_sq - ellipsoid plot

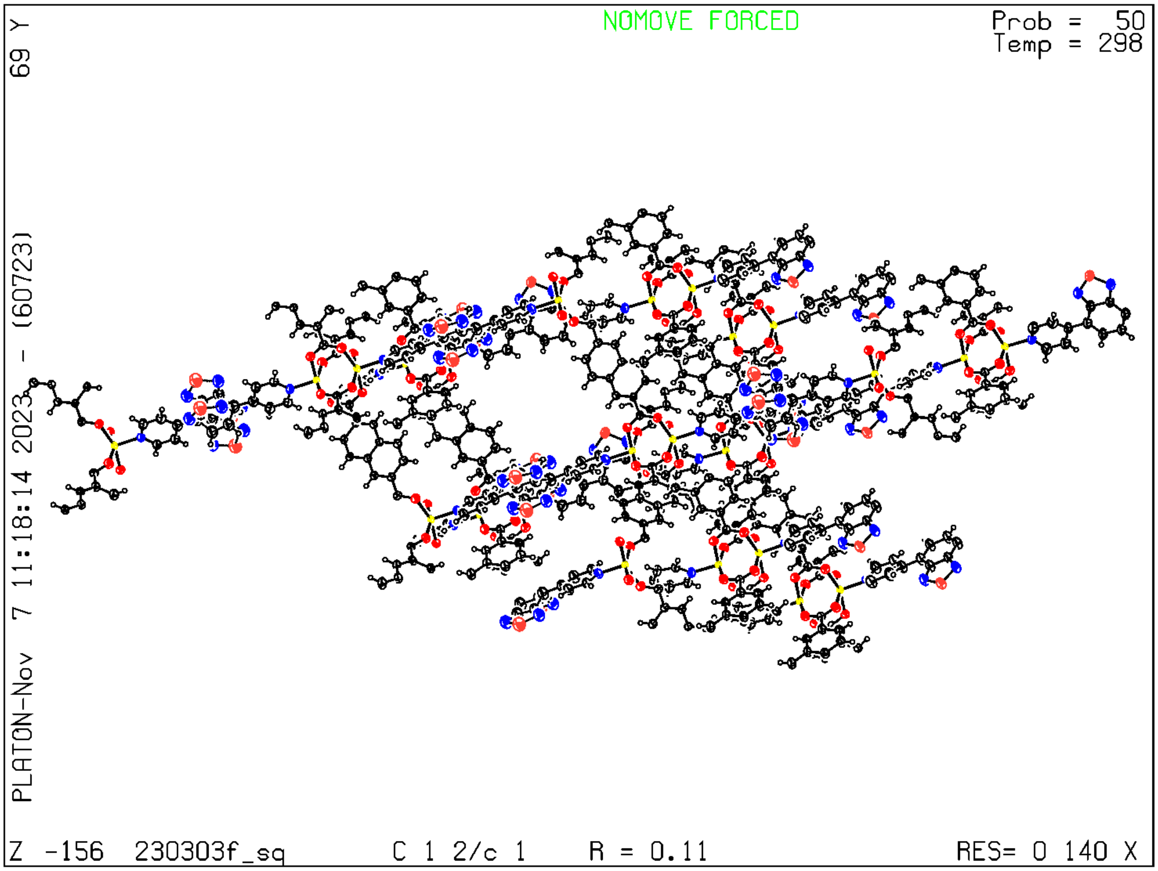

Supplement: Supplementary file 1 [file molecules-29-02755-s001.zip › checkcif-MOF-1.pdf]
