# Supplementary material for: A Benzothiadiazole-Based Zn(II) Metal–Organic Framework with Visual Turn-On Sensing for Anthrax Biomarker and Theoretical Calculation
Source: Molecules. 2024 Jun 9;29(12):2755. doi: 10.3390/molecules29122755 (PMC11206062; doi:10.3390/molecules29122755)
Supplement: Supplementary file 1 [file molecules-29-02755-s001.zip › Revised Supplementary material.pdf]

Figures

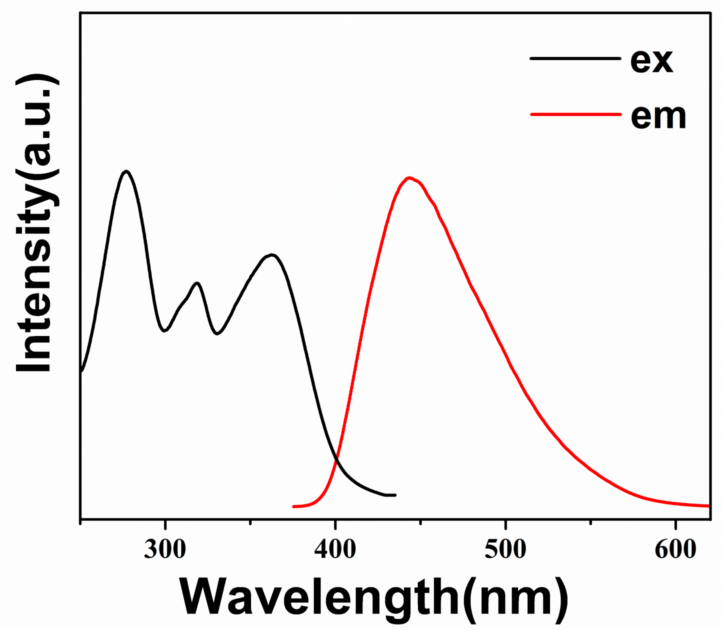

Figure S1 The excitation and emission of **MOF-1** in EtOH.

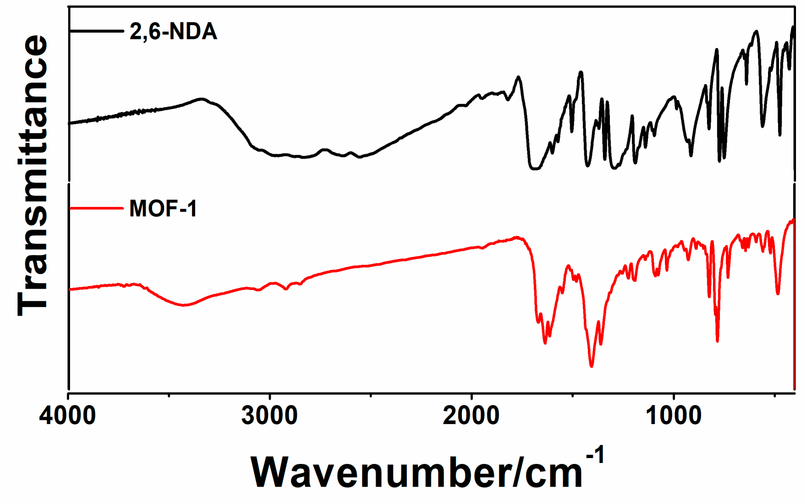

Figure S2. IR of **MOF-1** and organic ligand 2,6-NDA.

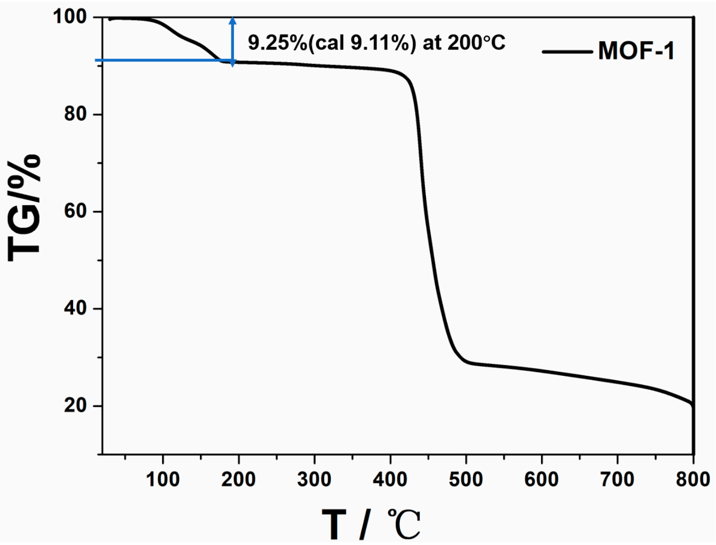

Figure S3. The TGA curve of **MOF-1** under N_2 atmosphere from 30 to 800 °C.

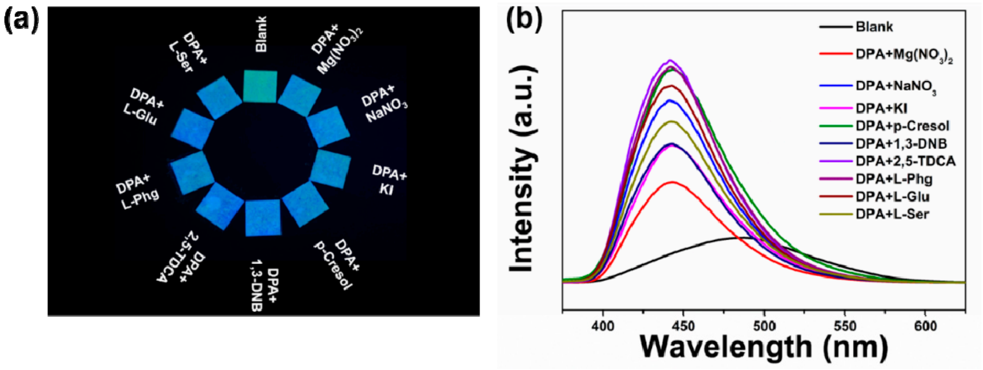

Figure S4. Interference experiments of different analytes with and without DPA-test strips (a) and the emission spectrum curves (b).

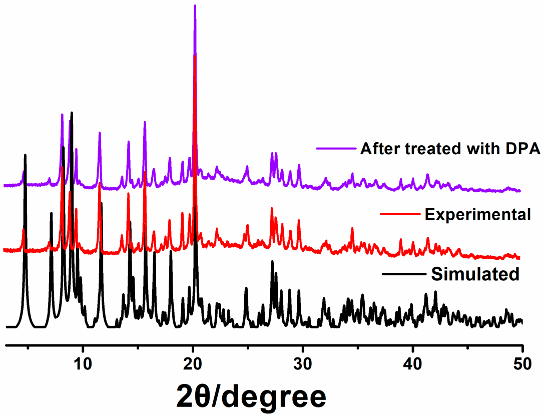

Figure S5 The PXRD pattern of **MOF-1-**and the sample after sensing DPA.

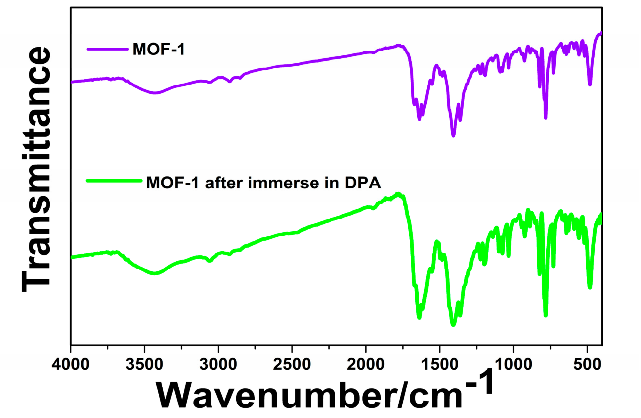

Figure S6 IR of **MOF-1** after sensing of DPA.

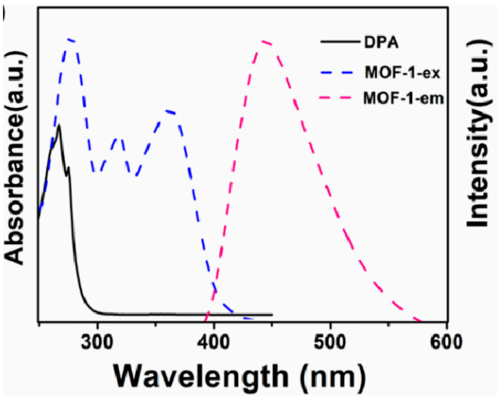

Figure S7 Absorption spectra of DPA and emission bands/excitation of **MOF-1**.

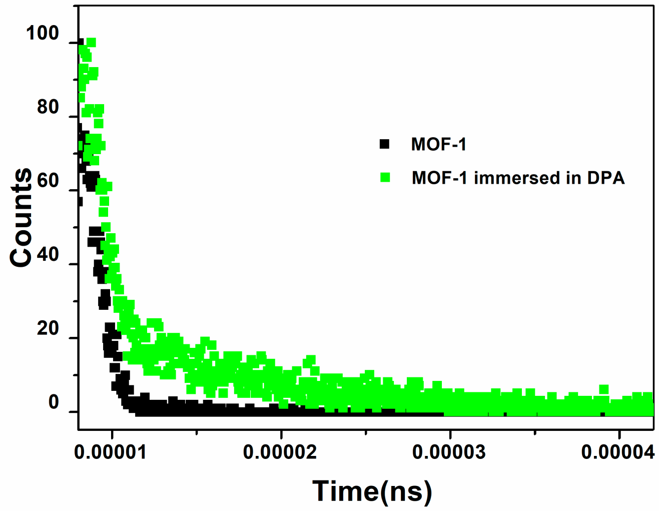

Figure S8 Fluorescence lifetime of **MOF-1** before and after DPA.

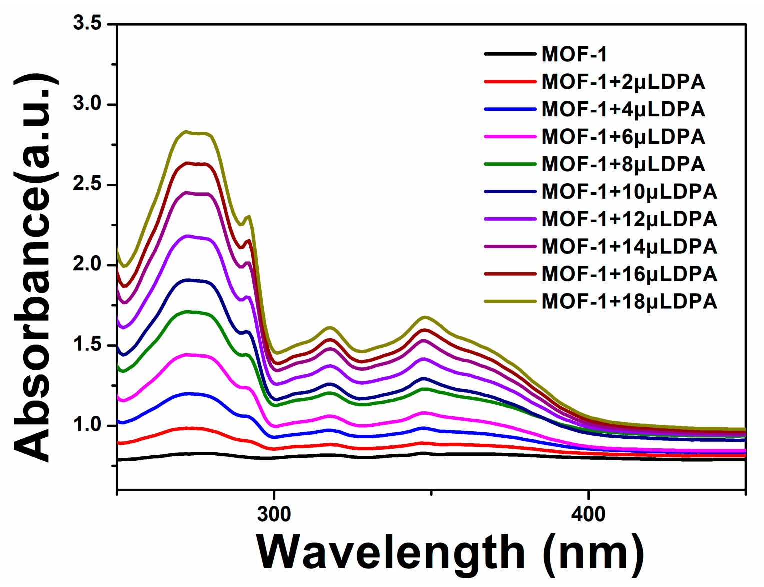

Figure S9 Absorption spectra of **MOF-1** dispersed in EtOH solution after adding different concentration of DPA.

Tables

Table S1 Selected bond lengths (Å) and angles (°) for **MOF-1.**

**Bond lengths (Å)**			
Zn1-O6	2.024(7)	Zn1-O7	2.043(7)
Zn1-N3	2.046(9)	Zn1-O3	2.050(7)
Zn1-O1	2.065(6)	Zn2-O4	2.041(7)
Zn2-O2	2.051(7)	Zn2-O5	2.061(7)
Zn3-N5	2.042(8)	Zn3-O12	2.037(7)
Zn3-O9	2.056(7)	Zn3-O11	2.050(6)
Zn3-O10	2.066(6)	Zn2-O8	2.065(6)
Zn2-N4	2.046(8)		
**Angles (º)**			
O3-Zn1-O1	88.3(3)	O5-Zn2-O8	88.1(3)
O4-Zn2-O2	88.4(3)	O4-Zn2-O5	89.2(3)
O4-Zn2-O8	159.5(3)	O4-Zn2-N4	102.3(3)
O2-Zn2-O5	157.5(3)	O2-Zn2-O8	86.3(3)
N4-Zn2-O2	105.2(3)	N4-Zn2-O5	97.1(3)
N4-Zn2-O8	98.2(3)	O11-Zn3-O10	86.9(3)
O12-Zn3-O11	87.6(3)	N5-Zn3-O10	97.9(3)
O12-Zn3-O9	87.0(3)	N5-Zn3-O11	103.4(3)
O12-Zn3-N5	101.3(3)	N5-Zn3-O9	98.4(3)

Table S2 Comparison of literature reports for MOFs as sensors of DPA.

Sensors	LOD	Medium	Ref
Zn-MOF	128nM	EtOH	[43]
Tb0.9Gd0.1-PBA	1.03μM	H_2O	[44]
Eu-MOF	0.41μM	H_2O	[45]
